# Kappa Opioid Receptors Reduce Serotonin Uptake and Escitalopram Efficacy in the Mouse Substantia Nigra Pars Reticulata

**DOI:** 10.3390/ijms24032080

**Published:** 2023-01-20

**Authors:** Alyssa M. West, Katherine M. Holleran, Sara R. Jones

**Affiliations:** Department of Physiology and Pharmacology, Wake Forest University School of Medicine, Medical Center Blvd, Winston Salem, NC 27157, USA

**Keywords:** serotonin, kappa opioid receptor, substantia nigra pars reticulata, serotonin release, serotonin uptake, serotonin transporter, fast-scan cyclic voltammetry, antidepressants, escitalopram, U50, 488

## Abstract

The serotonin and kappa opioid receptor (KOR) systems are strongly implicated in disorders of negative affect, such as anxiety and depression. KORs expressed on axon terminals inhibit the release of neurotransmitters, including serotonin. The substantia nigra pars reticulata (SNr) is involved in regulating affective behaviors. It receives the densest serotonergic innervation in the brain and has high KOR expression; however, the influence of KORs on serotonin transmission in this region is yet to be explored. Here, we used ex vivo fast-scan cyclic voltammetry (FSCV) to investigate the effects of a KOR agonist, U50, 488 (U50), and a selective serotonin reuptake inhibitor, escitalopram, on serotonin release and reuptake in the SNr. U50 alone reduced serotonin release and uptake, and escitalopram alone augmented serotonin release and slowed reuptake, while pretreatment with U50 blunted both the release and uptake effects of escitalopram. Here, we show that the KOR influences serotonin signaling in the SNr in multiple ways and short-term activation of the KOR alters serotonin responses to escitalopram. These interactions between KORs and serotonin may contribute to the complexity in the responses to treatments for disorders of negative affect. Ultimately, the KOR system may prove to be a promising pharmacological target, alongside traditional antidepressant treatments.

## 1. Introduction

Stress is widely considered to be one of the most common environmental risk factors for an array of psychiatric disorders [1,2,3]. Stress exposure has been shown to contribute to substance abuse, including triggering relapse in human and animal studies [4,5,6,7]. Additionally, a single traumatic stress exposure can be sufficient to cause depression or anxiety disorders [8,9,10]. Brain stress responses can include neuroinflammation [11,12,13,14] and alterations in key stress-related neuromodulators, such as the corticotropin-releasing hormone (CRH) and dynorphin, as well as mood and emotion regulators, such as dopamine and serotonin, to name a few [15,16]. To further complicate matters, in addition to changing their activity with stress, many stress-responsive neurotransmitters influence each other. For example, over the last decade, one cascade of events that occurs in response to stress has been studied in detail. After forced-swim stress, CRH is increased in the central amygdala, which then increases the activity of dynorphin neurons in multiple brain regions, and in the nucleus accumbens, elevated dynorphin inhibits dopamine release [17,18,19]. The sum of all these events produces a negative affective state in animals, which is known to contribute to anxiety-like, depression-like and low-motivation behavioral outcomes [17,20]. However, despite extensive research into the pathophysiology that links stress and psychiatric disorders, the circuitry and cellular mediators driving these interactions are not fully understood, and such knowledge gaps may obscure potential avenues to improve treatments for these disorders. The main purpose of the work described herein is to provide a better understanding of one specific interaction that is relatively understudied, between the serotonin system and the dynorphin/kappa opioid receptor (KOR) system.

KORs have been heavily implicated in a variety of disorders, including anxiety, depression, addiction, and pain [18,21,22,23,24,25,26,27]. As such, KOR ligands for the treatment of numerous disorders, such as pain and itch, are under development, including both newly synthesized and naturally bioactive compounds [28,29,30]. In the central nervous system, the KOR is involved in maladaptive stress responsivity, driving aversion, negative affect, and dysphoria in pain and stress models. This is thought to be due to KORs, found on monoaminergic neurons and axon terminals, which modulate dopamine, norepinephrine and serotonin signaling [19,31,32]. KOR modulation of dopamine has been heavily studied and many of the signaling pathways, as well as neurobiological and behavioral effects, are well known, including how KOR signaling can sometimes be both a consequence and a cause of stress exposure [33,34,35]. In particular, KOR inhibition of dopamine in the nucleus accumbens has been a focus in the field, due to the region’s involvement in stress, emotion, reward processing, and dysfunction in disorders of negative affect and addiction. Despite the serotonin system also being associated with stress responses and strongly linked to depression and anxiety disorders [36], our current knowledge of how the serotonin and KOR systems are connected is rudimentary. Opposing effects of acute and chronic kappa activation on serotonin activity have been observed [37,38]. Acute studies that examined direct KOR activation found decreased serotonin release and uptake in the dorsal and ventral striatum, similar to the effect of KOR activation on dopamine release in the ventral striatum [38]. Effects of short-term KOR activation on serotonin signaling have not yet been studied in other brain regions. A thorough understanding of the interactions of the serotonin and KOR systems throughout the brain may lead to better treatment options for disorders of negative affect.

Serotonin and KOR expression converge in numerous brain regions, one of the most substantial being the substantia nigra pars reticulata (SNr) [39]. The SNr is a part of the basal ganglia, an interconnected group of subcortical nuclei that control movement, as well as some cognitive and behavioral functions [39]. The SNr receives the densest serotonergic innervation in the central nervous system, and in this region, serotonin modulates both dopamine and GABA signaling [40]. SNr neurons travel to multiple brain regions and are heavily interconnected with the striatum, which in turn provides inhibitory input to the SNr via medium spiny neurons that express KORs and dopamine receptors [40]. The importance of understanding how serotonin in this region can be altered by KOR signaling is underscored by the fact that the SNr contains one of the greatest densities of KORs in the brain [40]. Furthermore, the SNr has recently been linked to negative affect-related behaviors, such as avoidance and drug reinstatement [41,42], highlighting the importance of studying the impact of KORs on serotonin signaling in the region. While KORs have been shown to modulate serotonin release and uptake in the striatum, their effects in the SNr are unknown [43,44,45]. A better understanding of how KORs modulate serotonin in the SNr may shed light on the role of this region in affective disorders.

In this study, we aimed to investigate whether KORs locally inhibit serotonin release and uptake in a manner that is similar to that which has been shown for KOR regulation of the dopamine system. A thorough understanding of the interactions between KORs and the serotonin system may better explain the role of KORs in affective disorders, particularly in the SNr, a region which may play a larger role in affective-related behaviors than previously thought. Therefore, the objective of this study was to examine the effects of acute KOR activation on serotonin release and uptake in this brain region, using fast-scan cyclic voltammetry (FSCV) with brain slices that contained the SNr. We first verified the measurement of serotonin in mouse brain slices using FSCV and isolated SERT activity using a new mathematical model. The activation of KORs with the agonist U50, 488 (U50) dramatically slowed serotonin uptake in a dose-dependent manner, as measured by the decreased maximal velocity of uptake through the serotonin transporter (SERT). It also decreased serotonin release in a dose-dependent manner, similar to the results previously observed in the striatum. We documented the dynamic time-dependent changes in serotonin release and uptake produced by the application of escitalopram, a selective serotonin reuptake inhibitor (SSRI). The peak serotonin signal amplitude was immediately increased by escitalopram, followed by a gradual decrease, but uptake was shown to continuously decrease across the hour-long time course of the experiment. These dynamic changes in serotonin release and uptake were fully or partially occluded when U50 was pre-applied to the slice. These results indicate that, in the SNr, the KOR inhibits the release and uptake of serotonin and reduces the efficacy of escitalopram. These findings underscore the importance of exploring the role of the KOR in serotonin signaling, as it pertains to the variability in antidepressant efficacy.

## 2. Results and Discussion

### 2.1. Ex Vivo Voltammetry Captures Dynamic Response of Serotonin to Escitalopram

**Figure 1:**
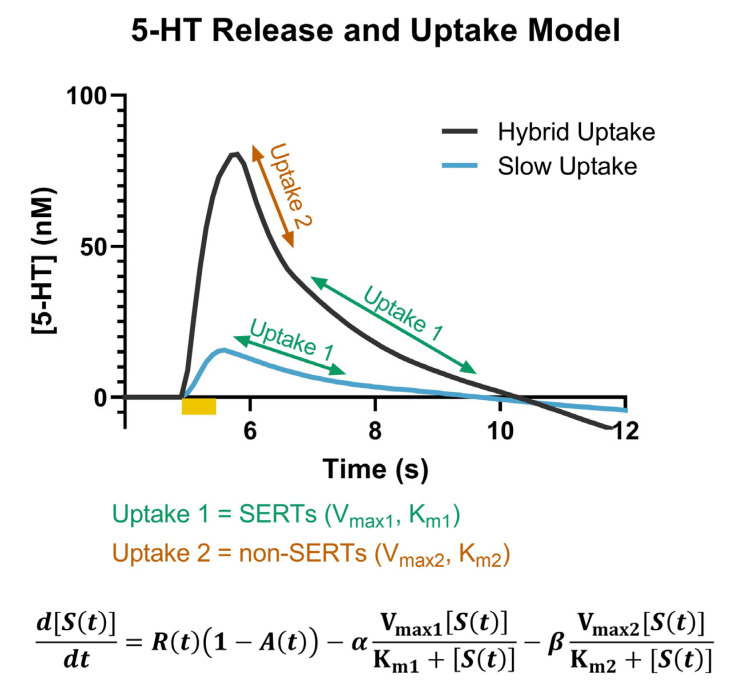
Representative model traces of the hybrid (dark grey) and slow (blue) uptake signals are shown. The portions of the curve associated with uptake 1 (SERTs) (green) and uptake 2 (non-SERTs) (orange) are noted. The full model that describes the serotonin concentration as a function of release, autoreceptors, and the two uptake mechanisms is included at the bottom of the figure.

Due to the dense innervation of serotonin in the SNr, we employed FSCV to measure the effects of KOR activation on serotonin signaling. Serotonin signals measured with FSCV can be distinguished based upon their characteristic oxidation peak at ~0.7 V [46,47]. They have also been established to have two distinct uptake mechanisms, which are differentiated based on the slope at which the signal returns to the baseline following stimulated release [48]. A “slow uptake” is attributable to uptake 1, or SERTs, which are high-affinity, but low-efficiency, transporters. “Hybrid uptake” has components of both uptake 1 and uptake 2, where uptake 2 is comprised of non-SERT monoamine transporters, e.g., organic cation transporter-3 (OCT3), which have low affinity but high efficiency, resulting in a faster uptake rate compared to uptake 1 [49]. In these experiments, both “slow” and “hybrid” uptake rates were measured in the SNr, as shown from the representative model traces in Figure 1. A third, “fast” uptake signal, which only includes uptake 2, has also been reported in the literature, but was not observed in any of the data analyzed herein [48]. The model presented in Figure 1 was initially described by Hashemi and colleagues and has since been incorporated into The Analysis Kid, an open-access kinetic modeling web application, which we used to determine SERT V_max_ and K_m_ (http://analysis-kid.hashemilab.com/ (accessed 8 September 2022)) [48,50].

**Figure 2:**
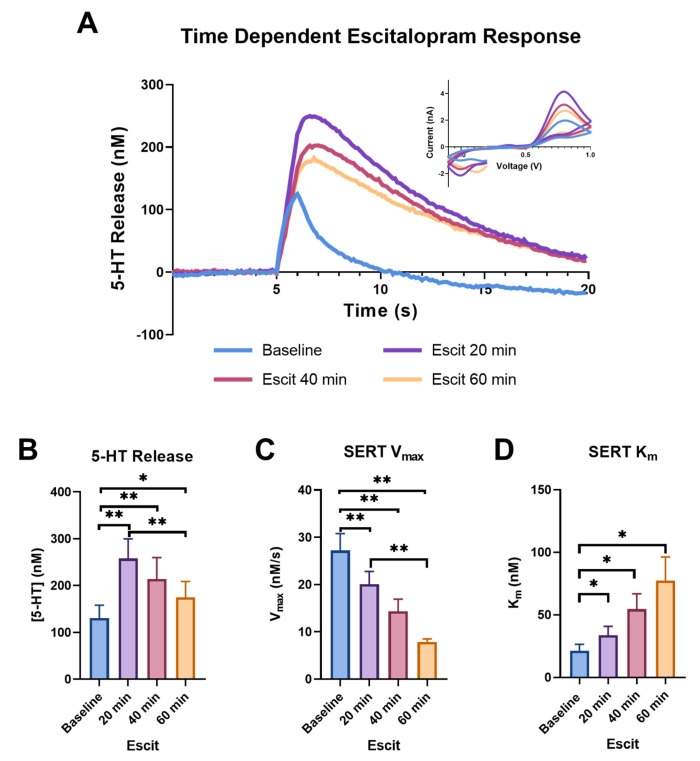
The concentration vs. time traces for the baseline (blue), 20 min (purple), 40 min (pink), and 60 min (light orange) following escitalopram (1 μM) are shown in (**A**), with the corresponding cyclic voltammograms in the inset. The peak height of the serotonin release is shown in (**B**) and the main effect of time was trending towards significance (ANOVA, 0.1 > *p* > 0.05). Significant increases for each time point are indicated on the graph (Tukey’s post hoc, * *p* < 0.05, ** *p* < 0.01). SERT V_max_ for each time point is shown in (**C**) and the main effect of time on V_max_ was recorded (ANOVA, *p* < 0.001). The significant effect of escitalopram on each time point is indicated on the graph (Tukey’s post hoc, ** *p* < 0.01). SERT K_m_ also demonstrated the main effect of time (ANOVA, *p* < 0.05), with individual time point differences shown in (**D**) (Tukey’s post hoc, * *p* < 0.05).

Figure 2 shows the dynamic changes in the release and uptake of serotonin caused by bath application of the selective serotonin reuptake inhibitor escitalopram (1 μM); we chose to highlight the baseline (blue), 20- (purple), 40- (pink), and 60-minute (light orange) time points from the hour-long period in which we monitored serotonin, following the application of escitalopram to the brain slice. In Figure 2A, the concentration of serotonin over time in response to electrical stimulation in the ex vivo brain slices that contained the SNr can be observed with the current vs. voltage traces shown in the inset. This cyclic voltammogram (CV) can be used as a “fingerprint” for serotonin with the presence of an oxidation peak at ~0.7 V [47]. Additionally, the changes in the signal amplitude and uptake following the application of escitalopram support the identification of the signal as serotonin [51].

The peak height was altered over time following escitalopram administration, with a trend towards significance (*p* = 0.0658, f = 35.43). A dramatic increase in peak height from the baseline was observed at the 20 min time point (baseline: 130.478 ± 27.692 nM; 20 min: 257.922 ± 41.780 nM; *p* = 0.0011). While the heights at the 40 and 60 min time points significantly increased from the baseline (40 min: 213.818 ± 45.755 nM, *p* = 0.0072; 60 min: 175.065 ± 33.550 nM, *p* = 0.0365), the height at the 60 min time point reduced compared to the 20 min time point (*p* = 0.0032) (Figure 2B). The increase in serotonin peak height following escitalopram is a phenomenon that is not fully understood. Previously, our lab has attributed an increase in peak height at the earlier time points to uptake inhibition, with the subsequent decrease at the later time points being a result of autoreceptor activation in response to the increase in extracellular levels at early time points, resulting in negative feedback, and subsequent decreased peak height at later time points. This phenomenon is extremely similar to dopamine release following cocaine application, a process we have extensively observed in our laboratory [52]. It has also been suggested that the increase in dopamine peak height following cocaine is due to a synapsin-dependent mechanism that increases exocytotic release [53]. As SERT has also been shown to interact with synapsin, this presents an additional possible mechanism for the increase in serotonin peak height following escitalopram [54,55]. Alternatively, previous studies that used ex vivo FSCV in cultured serotonin neurons did not observe this phenomenon and postulated that it is driven by factors within the non-serotonergic local environment (astrocytes, etc.), which are present in vivo and in slices, but lacking in cultured serotonin neurons [56].

We also found that escitalopram significantly slowed serotonin uptake. We observed this effect using the above-mentioned model, which fit the uptake curve to generate SERT K_m_ and showed significant slowing over the 60 min, as shown by a one-way repeated measure ANOVA (*p* = 0.0243, f = 10.61), followed by Tukey’s post hoc test, showing slowing at each point from the baseline (baseline: 21.282 ± 5.244 nM; 20 min: 33.687 ± 7.178 nM, *p* = 0.0163; 40 min: 54.618 ± 12.234 nM, *p* = 0.0396; 60 min: 77.411 ± 18.892 nM, *p* = 0.0463), as well as slowing from 20 min (40 min *p* = 0.0573; 60 min *p* = 0.0643) (Figure 2D). Escitalopram binds to the primary serotonin binding site of the SERT to competitively inhibit SERT activity [57]. Blocking this binding site results in an apparent change to K_m_, the binding affinity of the SERT for serotonin. 

Studies have also shown that escitalopram decreases serotonin uptake via a second mechanism, the internalization of SERT, which can be measured as a decrease in V_max_ [58,59]. We confirmed that SERT V_max_ continuously slows at 20, 40 and 60 min compared to the baseline (Figure 2C). An overall effect of time on the V_max_ was found using a one-way repeated measure ANOVA (*p* = 0.0007, f = 27.14), while a Tukey’s post hoc test found that all the time points were significantly slower compared to the baseline (baseline: 27.217 ± 3.558 nM/s; 20 min: 20.069 ± 2.701 nM/s, *p* = 0.0081; 40 min: 14.335 ± 2.574 nM/s, *p* = 0.0080; 60 min: 7.847 ± 0.641 nM/s, *p* = 0.0039), with the 60 min time point being significantly slower than the 20 min time point (*p* = 0.0099) and the 60 min time point being slower (*p* = 0.0635) than the 40 min time point. 

The dynamic changes to serotonin release and uptake when escitalopram is administered to ex vivo brain slices are consistent with previous work using in vivo fast-scan cyclic voltammetry, despite previously being reported to be exclusively observed using in vivo voltammetry [51]. However, we show here that ex vivo slice voltammetry can successfully capture these dynamic changes over time, indicating that these adaptations over time occur at the terminal level, which is not disrupted by the slice preparation process. 

### 2.2. KOR Activation Inhibits Serotonin Release and Uptake via SERTs

**Figure 3:**
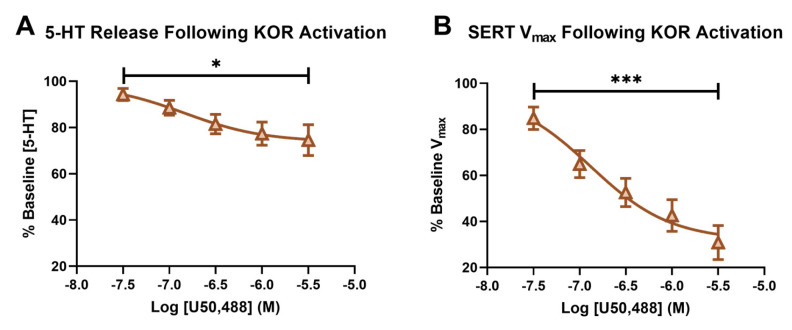
The percent baseline of the peak height following a concentration–response curve of U50 is shown in (**A**). The main effect of the concentration of U50 on the serotonin release (RM-ANOVA * *p* < 0.05) is shown. The percent baseline of serotonin V_max_ following a concentration–response curve of U50 is shown in (**B**), with the main effect of the concentration of U50 (RM-ANOVA, *** *p* < 0.001).

In Figure 3A, we demonstrated an inhibition of serotonin release in the SNr following the bath application of the KOR agonist, U50, causing the signals to decrease to 74.568% ± 6.637 of the baseline signal at the highest U50 concentration (3 μM). A repeated measures ANOVA revealed the main effect of the dose of U50 on the serotonin release (*p* = 0.0217, f = 5.226). The activation of KORs has been shown to result in reduced neurotransmitter release, due to terminal hyperpolarization via increased potassium channel conductance [60]. Previous work demonstrated that this effect includes serotonin, as acute activation of KORs reduced serotonin neuronal excitability in the dorsal raphe using whole-cell voltage-clamp electrophysiology [37]. Additionally, microdialysis studies found that U50 decreased serotonin in the dorsal raphe nucleus, median raphe nucleus, and nucleus accumbens core [61].

The effect of kappa activation on SERT function has been reported to be dependent on region, as well as short- or long-term, activation. While an increase in serotonin uptake rate and SERT surface expression was reported in whole brain synaptosomes following repeated KOR agonist administration [24], and also in the ventral striatum after long-term KOR activation via repeated stress [62], short-term KOR activation in synaptosomes from the dorsal and ventral striatum showed decreased SERT mediated uptake (V_max_) and SERT surface expression [38]. Using the model developed by Hashemi and colleagues, we were able to isolate SERT V_max_ and recapitulate these findings in the SNr, following short-term KOR activation for the first time [48]. Serotonin uptake, as measured by V_max1_ from the model described above, was significantly decreased, to 30.811% ± 7.418 from the baseline V_max1_ at the 3 μM concentration of U50. The main effect of the dose of U50 on serotonin V_max_ was confirmed using a repeated measures ANOVA (*p* = 0.0002, f = 34.84). (Figure 3B).

Similar to the serotonin release inhibition shown here, the inhibition of dopamine release by KORs is well documented and often associated with dysphoria [18,25]. Our data indicate that the regulation of serotonin by KORs in the SNr is two-fold, as the release of serotonin and SERT function are inhibited following U50. We, therefore, postulate that KOR modulation of serotonin contributes to dysphoria and negative affect. The recent link between the SNr and negative affect-related behaviors, such as avoidance and drug reinstatement [41,42], further supports this and highlights the significance of our findings that KORs modulate serotonin signaling within the region. 

### 2.3. Escitalopram Efficacy Altered by U50 Pretreatment

**Figure 4:**
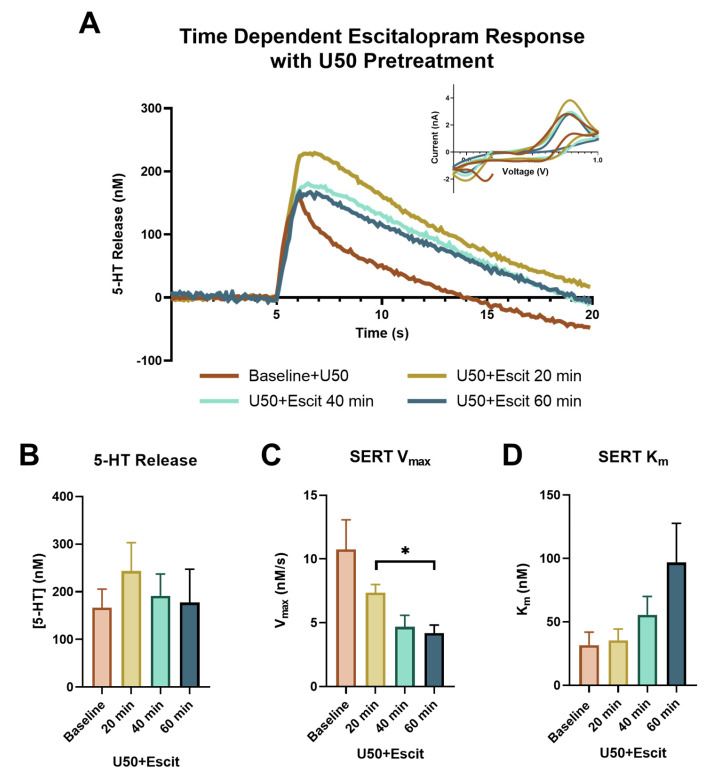
The concentration vs. time traces for the baseline (orange), 20 min (yellow), 40 min (light green), and 60 min (teal) following escitalopram (1 μM) are shown in (**A**), with the corresponding cyclic voltammograms in the inset. The peak height of the serotonin release is shown in (**B**), with no significant effects found. SERT V_max_ for each time point is shown in (**C**), with only one set of time points being significantly different, as indicated on the graph (Tukey’s post hoc, * *p* < 0.05). SERT K_m_, as shown in (**D**), demonstrated the main effect of time (ANOVA, *p* < 0.05).

To further assess the impact of KOR activation on SERTs, escitalopram was applied following the 3 μM concentration of U50, at the end of the concentration–response curve. In Figure 4A, the 20 min (yellow), 40 min (light green), and 60 min (teal) time points following escitalopram are highlighted and compared to the serotonin signal at the 3 μM dose of U50 (orange), just prior to adding escitalopram. The presence of 3 μM U50 on the slice markedly changed the impact escitalopram had on both the release and uptake of serotonin. The significant increase in peak height following escitalopram was noticeably absent in the presence of U50 (baseline: 166.364 ± 39.108 nM; 20 min: 243.333 ± 59.735 nM; 40 min: 191.414 ± 45.817 nM; 60 min: 177.374 ± 70.008 nM; *p* = 0.2015, f = 2.889) (Figure 4B). While the serotonin uptake still slowed, the dynamic response over time appeared to be lost (baseline: 10.747 ± 2.326 nM/s; 20 min: 7.364 ± 0.629 nM/s; 40 min: 4.685 ± 0.896 nM/s; 60 min: 4.187 ± 0.633 nM/s). The only significant difference in V_max_ was found at 60 min compared to 20 min, as shown by a one-way repeated measure ANOVA, followed by a Tukey’s post hoc test (*p* = 0.0189) (Figure 4C). The main effect of time on K_m_ following escitalopram was observed using a one-way repeated measure ANOVA (*p* = 0.0081, f = 12.77); however, a Tukey’s post hoc test did not reveal any significant differences between the individual time points (baseline: 31.703 ± 10.211 nM; 20 min: 35.422 ± 8.962 nM; 40 min: 55.564 ± 14.544 nM; 60 min: 96.882 ± 30.817 nM). This indicated that the dynamic effect of escitalopram on the apparent K_m_ value was also blunted in the presence of KOR activation (Figure 4D).

**Figure 5:**
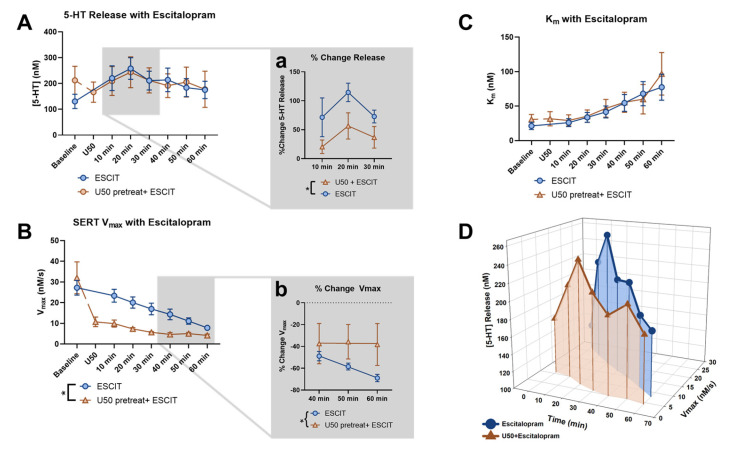
The effect of escitalopram on the peak height of serotonin release across all time points was compared for the escitalopram-alone group (blue) and the U50 pretreatment followed by escitalopram group (orange) in (**A**). Analyzing the last file before and following escitalopram application revealed the significant effect of time (2-way ANOVA, *p* < 0.05). The inset (**Aa**) focuses on the percent change in the first half of the time course and shows the significant effect of the groups (2-way ANOVA, * *p* < 0.05). The effect of escitalopram on the V_max_ of SERT across all time points was compared for the two groups in (**B**). Analyzing the last file before and following escitalopram application revealed the significant effect of time (2-way ANOVA, *p* = 0.0001) and the groups (2-way ANOVA, *p* < 0.05), along with the interaction effect (2-way ANOVA, *p* < 0.01). The inset (**Bb**) focuses on the percent change in the last half of the time course and shows a significant difference in the linear regression of the two groups (* *p* < 0.05). The effect of escitalopram on the K_m_ of SERT across all time points was compared for the two groups in (**C**), with no significant differences found. (**D**) presents the changes in the peak height of serotonin release and SERT V_max_ as a 3-dimentional plot over the time course, following the application of escitalopram for each group.

To examine the effects of KOR activation on escitalopram-induced serotonin signals, we initially compared raw serotonin peak heights for the escitalopram-alone group and the U50 pretreatment followed by escitalopram group at each ten-minute collection for an hour. An ANOVA, with time and group as factors, demonstrated the main effect of time (*p* = 0.0235, f = 5.312), but no significant main effect of the groups (*p* = 0.9803, f = 0.0006357) (Figure 5A) was recorded. However, since the U50 pretreatment caused the peak height to decrease from the original baseline, we examined the percent change with respect to the last file collected prior to escitalopram application and found the groups to be significantly different at the beginning of the time course, from 10 to 30 min (*p* = 0.0424, f = 5.267) (Figure 5a).

In Figure 5B, we also compared the V_max_ raw values over the time courses and found that they were dramatically different with regard to the groups (*p* = 0.0277, f = 6.425) and time (*p* = 0.0001, f = 20.45) and also had a significant interaction effect (*p* = 0.0020, f = 3.992). The substantial reduction in V_max_ by U50 from the original baseline resulted in the response to escitalopram to appear blunted (baseline: 32.019 ± 7.703 nM/s; U50: 10.7147 ± 2.326 nM/s; *p* = 0.0499). This blunted response was the most apparent at the later time points, as shown by plotting the percent change in V_max_ induced by escitalopram (Figure 5b). A flattening of the curve at the 40–60 min time points can be observed with a linear regression, such that the elevations, or intercepts, of the lines were significantly different (*p* = 0.0283, f = 5.327). This indicates that the V_max_ of the U50 pretreatment group reached an asymptote after roughly 30 min, while the escitalopram-alone group was still decreasing throughout the time course. Ultimately, the two groups appeared to converge at a similar maximal reduction point in V_max_, indicating that U50 and escitalopram employ the same mechanisms to reduce V_max_, and prior U50 treatment occluded some of escitalopram’s effects. Indeed, previous work has shown that both U50 and escitalopram cause a decrease in SERT surface expression, which can be observed as a decrease in V_max_. However, the asymptote for V_max_ was not at zero, suggesting that a certain number of functional SERTs are maintained at the surface of the membrane, regardless of the pharmacological insult. 

The apparent K_m_ significantly increased with escitalopram over time for both groups (*p* = 0.0003, f = 17.20), and there was no difference between groups (*p* = 0.7464, f = 0.11) (Figure 5C). This suggests that the change in the serotonin signal following U50 pretreatment can be primarily attributed to the effect U50 has on the release of serotonin and V_max_, and the relationship between the two can be observed throughout the time course in Figure 5D. 

These findings suggest that states of short-term enhanced KOR activation, such as acute stress exposure, may result in the decreased efficacy of escitalopram. However, previous examinations of the effects of pharmacological and behavioral activation of KOR on SERT surface expression have proven to be heavily dependent on the cellular and environmental context. The variation in previous findings suggests that the interactions between the serotonin and kappa systems are complex, varying by short- and long-term KOR stimulation, as well as the brain region being examined. For instance, long-term KOR activation via repeated swim-stress exposure induced increased SERT expression in the ventral striatum, but not the dorsal striatum, hippocampus, prefrontal cortex, amygdala, or dorsal raphe [62]. Additionally, an increase in the serotonin uptake rate and SERT surface expression was observed in whole brain synaptosomes following repeated KOR agonist administration [24]. However, short-term KOR activation from direct application of a KOR agonist onto synaptosomes from the dorsal and ventral striatum showed decreased SERT-mediated uptake (V_max_) and SERT surface expression [38]. Due to the intricacy of the KOR and serotonin interactions, we believe it is important to first understand the acute effects of KOR stimulation on serotonin signaling. This study closely mirrors the work by Ramamoorthy and colleagues, in that we also examine the acute application of a KOR agonist to naïve animals [38]. A decrease in SERT surface expression would be understood as a decrease in V_max_, as observed in previous studies [63]. Likewise, a decrease in SERT surface expression explains the loss of dynamic changes in the uptake of serotonin when escitalopram is applied, as the change in uptake following escitalopram is due, in part, to the internalization of the transporter. A decrease in SERT surface expression would occlude the subsequent SERT internalization caused by escitalopram. Although studies by Chavkin and colleagues have shown long-term KOR stimulation to have opposite effects, resulting in an increase in SERT efficiency [24,62], it is plausible to assume that the efficacy of escitalopram will still be impacted. Future research that focuses on long-term KOR activation, in combination with escitalopram administration, is necessary to determine the extent to which SSRI efficacy may be altered, particularly in stress or depression models. Nonetheless, these findings are an important step in fully understanding KOR/serotonin interactions, particularly as they may alter the efficacy of SSRIs, such as escitalopram, in individuals with augmented KOR function, which is likely to be highly comorbid for those with depression and anxiety-related disorders. This line of research is particularly important, as it may offer a new pharmacological target to be used in combination with SSRIs to improve patient outcomes.

## 3. Materials and Methods

### 3.1. Animals

Male C57BL/6J mice (*n* = 5–7 per group, 6–12 weeks old, Jackson Laboratories) were used for the experiments. All animals were subjected to a 12:12 light cycle (lights on at 0700) and given access to standard rodent chow and water ad libitum. All experiments were performed during the animals’ light cycle. Animal care, handling, and experimental protocols were approved by the Wake Forest School of Medicine Institutional Animal Care and Use Committee and in accordance with all the National Institutes of Health Animal Care Guidelines.

### 3.2. Brain Slice Preparation

The animals were deeply anesthetized using isoflurane gas in an induction chamber, prior to being rapidly decapitated. The brains were removed and placed into ice cold, pre-oxygenated artificial cerebrospinal fluid (aCSF; 126 mM of NaCl, 2.5 mM of KCl, 1.2 mM of NaH_2_PO_4_, 1.4 mM of CaCl_2_, 2.4 mM of MgCl_2_, 25 mM of NaHCO_3_, 11.0 mM of glucose and 0.4 mM of L-ascorbic acid) [64]. A vibratome (Leica Biosystems, Buffalo Grove, IL, USA) was used to prepare the coronal brain slices (300 μm thick) that contained the SNr. The brain slices were transferred to recording chambers and incubated at 32 °C in oxygenated aCSF for at least one hour prior to the experiment. 

### 3.3. Fast-Scan Cyclic Voltammetry

Carbon fiber microelectrodes (CFMs) were prepared in-house using glass capillaries (1.2 mm × 0.68 mm, A-M Systems, Sequim, WA, USA) and carbon fibers (100–150 μm, Goodfellow Corp., Berwyn, PA, USA) [64]. The CFMs were placed in close proximity to a bipolar-stimulating electrode (Plastics One, Roanoke, VA, USA) on the surface of the slices in the SNr. A serotonin-specific waveform (0.2 V to 1.0 V to −0.1 V to 0.2 V vs. silver/silver chloride, 1000 Vs^−1^) was applied to the working electrode at a frequency of 10 Hz and changes in the current at the oxidation potential value for serotonin (~0.7 V) were monitored. Each file had a duration of 20 s with stimulation (30 pulses, 30 Hz, 350 μA, 4 ms) applied to elicit serotonin release at 5 s, and a period of ten minutes was allowed between stimulation trains [52]. All the files were collected and analyzed with Demon Voltammetry and Analysis software [65]. Once a stable baseline was achieved, one drug group was established, which consisted of slices that received a bath application of 1 μM of escitalopram oxalate (Sigma, Burlington, MA, USA), after which changes in the serotonin signal were monitored for 60 min. In the second drug group, the slices received a bath application of U50 (National Institute on Drug Abuse Drug Supply) according to a half-log scale to generate a concentration–response curve (30 nM–3 μM, 40 min at each dose) and following the last dose, 1 μM of escitalopram (60 min) was administered. The electrodes were post-calibrated in a 3 μM solution of serotonin in aCSF, using a custom-built flow-injection system. The calibration factors for over 50 electrodes were determined and averaged together to generate the calibration factor.

### 3.4. Data Analysis and Statistics

Data were analyzed using the FSCV Analysis tool and two reuptake analyses within The Analysis Kid web application. The terms used for the two reuptake analyses were informed by those previously determined in the SNr by Hashemi and colleagues [66]. Statistical analysis and graph preparation of the μM release, V_max_, and K_m_ were carried out using GraphPad Prism (v.8, La Jolla, CA, USA). A one-way repeated measures analysis of variance (ANOVA), followed by a Tukey post hoc analysis, were used to determine differences in the release of serotonin, V_max_, and K_m_. In the case of excluded values within a group, the data were instead fitted using a mixed model. To determine if the dose of U50 had a significant effect on the percent baseline of release or half-width, a repeated measures ANOVA was applied. When comparing the escitalopram-alone and U50 pretreated with escitalopram groups, a two-way ANOVA, with group and time as factors, was used to determine the differences in the percent change in the release of serotonin or V_max_. In the event of excluded values, a mixed model was instead applied. The Sidak’s post hoc test was used to determine specific differences. Each group had 5–7 animals, with 1–2 slices used from each animal. The signals that did not change when escitalopram was applied were excluded, in addition to the signals in which a stimulation artifact exceeded the amplitude of the signal at any point throughout the experiment.

## 4. Conclusions

These studies demonstrated that serotonin can be measured through ex vivo in slice preparation and were capable of capturing the dynamic changes to both the release and uptake of serotonin in response to escitalopram, which were previously thought to be limited to in vivo studies using intact tissue. We also show for the first time that in the SNr, serotonin release and uptake are modulated by KOR activation. Furthermore, KOR activation results in changes to V_max_, which prevent the dynamic changes previously observed in naïve tissue when escitalopram was applied to the slice. Previous studies in other brain regions also observed a decrease in V_max_ and confirmed a reduction in SERT surface expression [38]. A decrease in surface SERTs following U50 explains why escitalopram failed to decrease V_max_ in the same manner as in naïve animals. These results indicate that acute KOR activation has profound effects on serotonin uptake and can limit the effectiveness of escitalopram. While these effects may be different during chronic KOR activation, acute KOR activation states, such as stress or pain, can have significant implications for people prescribed escitalopram or other antidepressants, as it may limit their effectiveness. Modulation of serotonin via KORs may, in fact, provide a possible reason for the variability in efficacy observed in patients who are prescribed antidepressants, and ultimately provide a new pharmacological target to be used in combination with the current treatment approaches. The findings we present herein lay the groundwork for future studies to focus on the combined administration of KOR antagonists and SSRIs in the case of acute KOR activation. Additionally, they highlight the importance of studying KOR and serotonin interactions in the context of affective disorders and how they may limit or alter SSRI efficacy. 

## Data Availability

The data presented in this study are openly available in the Open Science Framework at osf.io/gnzvb.
